# SIV Evolutionary Dynamics in Cynomolgus Macaques during SIV-*Mycobacterium tuberculosis* Co-Infection

**DOI:** 10.3390/v14010048

**Published:** 2021-12-29

**Authors:** Kaho H. Tisthammer, Christopher Kline, Tara Rutledge, Collin R. Diedrich, Sergio Ita, Philana Ling Lin, Zandrea Ambrose, Pleuni S. Pennings

**Affiliations:** 1 Department of Biology, San Francisco State University, San Francisco, CA 94132, USA; pennings@sfsu.edu; 2 Department of Microbiology and Molecular Genetics, University of Pittsburgh School of Medicine, Pittsburgh, PA 15219, USA; cjk14@pitt.edu (C.K.); zaa4@pitt.edu (Z.A.); 3 Department of Pediatrics, University of Pittsburgh School of Medicine, Pittsburgh, PA 15224, USA; tar36@pitt.edu (T.R.); crd64@pitt.edu (C.R.D.); philana.lin@chp.edu (P.L.L.); 4 Center for Vaccine Research, University of Pittsburgh School of Medicine, Pittsburgh, PA 15261, USA; 5 Abintus Bio, Inc., San Diego, CA 92121, USA; sergioita@gmail.com

**Keywords:** SIV, co-infection, viral evolution, Mtb, viral diversity, macaques

## Abstract

Co-infection with *Mycobacterium tuberculosis* (Mtb) and human immunodeficiency virus (HIV) is a worldwide public health concern, leading to worse clinical outcomes caused by both pathogens. We used a non-human primate model of simian immunodeficiency virus (SIV)-Mtb co-infection, in which latent Mtb infection was established prior to SIV_mac251_ infection. The evolutionary dynamics of SIV *env* was evaluated from samples in plasma, lymph nodes, and lungs (including granulomas) of SIV-Mtb co-infected and SIV only control animals. While the diversity of the challenge virus was low and overall viral diversity remained relatively low over 6–9 weeks, changes in viral diversity and divergence were observed, including evidence for tissue compartmentalization. Overall, viral diversity was highest in SIV-Mtb animals that did not develop clinical Mtb reactivation compared to animals with Mtb reactivation. Among lung granulomas, viral diversity was positively correlated with the frequency of CD4+ T cells and negatively correlated with the frequency of CD8+ T cells. SIV diversity was highest in the thoracic lymph nodes compared to other sites, suggesting that lymphatic drainage from the lungs in co-infected animals provides an advantageous environment for SIV replication. This is the first assessment of SIV diversity across tissue compartments during SIV-Mtb co-infection after established Mtb latency.

## 1. Introduction

Nearly 38 million people worldwide are infected with HIV and they are 20 times more likely to become ill with tuberculosis (TB) than uninfected individuals [1]. Indeed, co-infection with *Mycobacterium tuberculosis* (Mtb), the etiologic bacterium that causes TB, is the leading cause of death among people living with HIV [1]. While the complex synergy between HIV and Mtb is not well understood, it is known that infection with one pathogen accelerates the progression of the other [2]. One reason that Mtb could accelerate HIV progression is because HIV/Mtb co-infected individuals have higher viremia [3] and within-host HIV diversity in the plasma [4,5] compared to individuals infected with HIV only. HIV diversity in turn plays a key role in disease progression, as higher diversity can lead to the generation of mutants that result in viral immune escape and resistance to drugs. Higher plasma viremia and faster disease progression are also known to be associated with higher viral diversity [6,7,8,9,10]. Conversely, HIV is the most common risk factor to progression to active TB, which can occur after primary Mtb infection or long-standing controlled latent Mtb infection, and TB is associated with higher death rates among those with HIV [11]. It is difficult to study how the two pathogens interact in the same host, particularly as human studies are often limited to cross sectional cohort investigations (i.e., a single time point) where it is unclear how long patients have been infected with HIV or Mtb and which infection occurred first.

Given these limitations of human studies and the need to address more mechanistic questions, animal models have been developed to recapitulate these infections. Simian immunodeficiency virus (SIV) and Mtb infections in macaques mimic both HIV and Mtb pathogenesis and immunity observed in humans, respectively [12,13]. We use this co-infection model as it offers a controlled system for detailed study of how Mtb affects viral populations in both the blood and tissues at specific time points after defined viral and bacterial infection, which cannot be achieved in human subjects. Importantly, this study investigates a clinical scenario in which Mtb infection is first established and controlled by the immune system (termed latent infection) in the lungs and the thoracic lymph nodes (LNs) prior to infection with SIV.

Our previous work on SIV in macaques has shown different population dynamics between anatomic compartments within macaques [14,15]. This suggests that viral evolution, including immune escape and development of drug resistance, may depend on interactions between partially independent tissues, some of which have viral populations that are more or less isolated from the rest of the body, different tissue resident immune responses, and/or drug pressure. Here, we are interested to determine whether TB granulomas could also harbor isolated SIV populations and if viral divergence occurs within these sites.

Granulomas are the histopathologic hallmark of TB that are comprised of highly organized collections of immune cells that function to contain Mtb. We have previously shown that individual granulomas each have an independent microenvironment with different bacterial burden and immune responses from each other, even in the same lung lobe of the same host [16]. These immune cells could be an important reservoir for HIV. Specifically in the case of HIV/Mtb co-infection, one study compared the inter-compartmental HIV diversity between plasma and rare TB spinal granulomas [17]. In four of the six patients, distinct monophyletic populations were observed in the spinal granulomas when compared to plasma, suggesting that viral divergence and evolution can occur within these tissue compartments. However, spinal granulomas are an uncommon manifestation of TB and it remains unclear if the lung and thoracic LN, the most common sites of TB granulomas, show viral divergence and evolution.

There is a long tradition of studying evolution of SIV in macaques as a model system for HIV infection. For example, a study by Burns and Desrosiers is an early description of within-host evolution of a SIV strain in rhesus macaques [18]. The envelope glycoprotein (Env) of SIV or HIV interacts directly with CD4 and co-receptors on target cells for virus entry and is the primary target of host antibody responses. Thus, it plays a crucial role in viral immune evasion. Previous studies have reported robust antibody responses to Env in macaques when infected with pathogenic SIV strains [19,20]. High variation and evidence of positive selection in the V1 loop of the Env surface subunit (gp120) of SIV have been reported [19,20,21]. Overbaugh et al. studied SIV env evolution in two pigtailed macaques that developed AIDS after infection with SIV [22]. Both of these studies focused on the env gene and found a variety of amino acid substitutions in the Env V1 and V4 loops. Burns and Desrosiers also found several deletions near the V4 loop in one of the two monkeys studied [18]. Sato et al. analyzed SIV in a single animal and found strong evidence for selection of several non-synonymous escape mutants in env [20]. This detailed study included phenotypic analysis of individual mutations. A recent study also showed dynamic evolution of insertion or deletion mutations (indels) in SIV env [19].

In this study, we specifically assessed the V1-V2 loop region of Env in cynomolgus macaques either infected with SIV_mac251_ alone or with latent Mtb infection and subsequently infected with SIV_mac251_ [23]. Among the co-infected animals, SIV infection led to Mtb reactivation in 60% of animals, defined as observed evidence of new TB disease progression after SIV infection. Our goal was to investigate divergence and diversity of the virus in the blood and several different tissue compartments relevant to TB among animals infected with SIV only or with Mtb co-infection with or without evidence of reactivation.

## 2. Materials and Methods

### 2.1. Experimental Design

Tissues used for this study came from a previously published investigation of seven adult cynomolgus macaques (*Macaca fasicularis*) with latent Mtb infection. These animals were initially infected with low dose (~15 colony forming units) Mtb (barcoded Erdman strain) [24] via bronchoscopic instillation and followed microbiologically, clinically, and radiographically. Clinically latent Mtb infection in this model was defined at 6 months post-infection as a lack of clinical signs of tuberculosis disease or microbiologic evidence of Mtb shedding [23]. These macaques were subsequently infected intravenously with 1.67 × 10^5^ viral RNA copies of SIV_mac251_ (provided by Dr. Keith Reimann, Beth Israel Deaconess Medical Center, Harvard University) and followed for an anticipated 8 weeks. Mtb reactivation was defined as radiographic evidence of newly disseminated granulomas after SIV infection, as measured by positron emission tomography and computed tomography and previously published [23]. Mtb reactivation was observed in four of the seven animals. In some cases, animals that developed clinical signs of deterioration (Animal IDs 16314, 20615, Table S1) were euthanized prior to the planned 8 weeks of follow-up.

**Figure 1:**
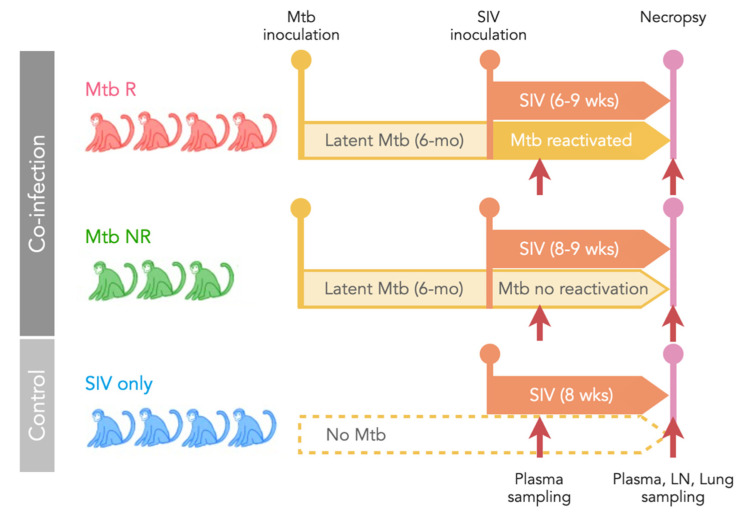
Study design. Seven cynomolgus macaques with latent Mtb infection (for at least 6 months) were subsequently infected with SIV_mac251_. Reactivation of Mtb occurred in four animals (Mtb R) and Mtb did not reactivate in three animals (Mtb NR). A control group of four animals was infected only with SIV (SIV only). Plasma samples were collected at 2–3 weeks post-SIV infection and both plasma and tissues were collected at necropsy (6–9 weeks post-SIV infection).

In addition to the seven Mtb-infected animals, four adult cynomolgus macaques without Mtb infection were infected with SIV_mac251_ in the same manner to serve as a control group. The animals were divided into three groups: (1) animals with Mtb reactivation (Mtb R, *n* = 4), (2) animals without Mtb reactivation (Mtb NR, *n* = 3), and (3) SIV only controls (SIV only, *n* = 4) (Figure 1, Table S1). Plasma samples were collected 2–5 weeks after SIV infection and plasma and tissue samples were collected at the time of necropsy (nex; 6–9 weeks after SIV infection). Viral RNA from plasma, peripheral lymph nodes (LN), and different parts of thoracic LNs and lungs, including granulomas, were analyzed in this study (see Table S1 for details). Viremia/RNA copy numbers and CD4+ T-cell counts and proportions in the samples were previously published [23] (Figure 1, Figure 4 and Figure 6A) and are listed in Table S2.

### 2.2. Ethics Statement

All protocols and procedures performed on animals were approved by the University of Pittsburgh Institutional Animal Care and Use Committee (IACUC, approval number 17050656) and consistent with the national guidelines established in the Animal Welfare Act and Guide for the Care and Use of Laboratory Animals as mandated by the U. S. Public Health Service Policy (PHS, PHS policy D16–00118).

### 2.3. Sequencing

RNA was extracted from the challenge virus stock, plasma, and tissues as previously published [23]. MiSeq sequencing was performed using previously published conditions [25]. Briefly, synthesis of cDNA was performed using up to 10,000 viral RNA copies and a primer complementary to the *env* V3 region (5′-GTGACTGGAGTTCAGACGTGTGCTCTTCCGATCTCAGTAAGTCTGTGTCTCCATCATCCTTGTG-3′) with SuperScript III reverse transcriptase (Thermo Fisher) and was then purified using the Agencourt RNAClean XP kit (Beckman Coulter). Preparation of the MiSeq library was performed using two PCR reactions. The first reaction was performed using the KAPA2G Robust system (Roche) with Mac251V1-F (5′-GCCTCCCTCGCGCCATCAGAGATGTGTATAAGAGACAGTACCAGCTTGGAGGAATGCGAC-3′) and ADPT-2a-R (5′-GTGACTGGAGTTCAGACGTGTGCTC-3′) primers. The PCR conditions were 95 °C for 1 min; 25 cycles of 95 °C for 15 s, 58 °C for 1 min and 72 °C for 30 s; and 72 °C for 3 min. PCR products were purified using the AMPure XP PCR cleanup kit (Beckman Coulter). The second PCR reaction was performed using the KAPA HiFi kit (Roche) with forward (5′-AATGATACGGCGACCACCGAGATCTACACGCCTCCCTCGCGCCATCAGAGATGTG-3′) and reverse (5′-CAAGCAGAAGACGGCATACGAGATNNNNNNGTGACTGGAGTTCAGACGTGTGCTC-3′) primers, with 6 Ns representing the position of 24 Illumina bar codes. The PCR conditions were 95 °C for 2 min; 25–35 cycles of 98 °C for 20 s, 63 °C for 15 s and 72 °C for 30 s; and 72 °C for 3 min. The second-round PCR products were purified using the QIAquick gel extraction kit (Qiagen). PCR products were quantified using the Qubit dsDNA HS Assay (Thermo Fisher) and were pooled in equal amounts. The pooled libraries were purified using the AMPure XP PCR cleanup kit. Libraries were sequenced using the Illumina MiSeq system with 300 nucleotide paired-end sequencing.

### 2.4. Sequence Assembly and Variant Calling

After removing the adapters and PhiX control library, all bases with a phred score < 30 were trimmed from both ends using BBTools [26]. All reads were then mapped to the *env* sequence of the SIV_mac251_ reference genome (MK686248) using bwa v.0.7.17 [27]. Consensus sequences for each sample were created in Geneious 11.1.4 (Biomatters Ltd. Auckland, New Zealand), and all reads were mapped again to its own consensus sequence using bwa. The generated sam files were converted to bam files using samtools [28], which were converted to frequency tables of nucleotides (nt) at each position using Rsamtools [29] in R v. 4.1.0 [30]. After trimming, the sites included in our analyses ranged from amino acid (AA) 32 (nt94) to AA272 (nt825). For each sample, sites with <100 read counts were removed from subsequent analyses. Due to low quality towards the end of reads in some of our samples (including the challenge stock), forward and reverse reads did not merge after trimming, creating a missing region in the center that corresponds to the variable V1 loop. Therefore, we excluded the missing region (AA130–177), leaving us with 585 sites for the average diversity calculation.

The frequency tables were further processed to calculate transition and transversion mutation frequencies based on (1) the consensus of the stock virus (i.e., divergence) at each site, and (2) the majority nucleotides of each sample (i.e., diversity). The types of mutations (whether the mutations result in synonymous or nonsynonymous changes) at each site and amino acid residues at each codon were also identified. We used SIV_mac239_ (M33262) positioning to describe all AA positions and SIV_mac251_ (MK686248) for nt positions.

### 2.5. Immunology

Flow cytometry of lung granuloma samples was performed in a previously published study [23]. Briefly, after harvesting samples of interest at necropsy (e.g., lung, lung granuloma, lymph node), tissues were homogenized into single cell suspensions. Cells were then stained for frequency (CD3, CD8, CD4) and function (granzyme B, IFN-g, IL-2, TNF, IL-17) of CD4+ and CD8+ T cells [23].

### 2.6. Statistical Analysis

Statistical analyses were conducted using non-parametric methods. We calculated the diversity levels of each sample as mean percent minor variant frequency. Using these values as diversity for each sample, we compared means using the Mann–Whitney *U* test, with *p*-values corrected for multiple pairwise comparisons (‘adjusted *p*-value’) with the Holm method. For associations, the Spearman’s rank correlation was used. A generalized linear mixed model (GLMM) was used to assess the factors affecting viral diversity levels using the glmmTMB package (Brooks et al., 2017). The best model was selected based on the AIC scores. The Wald Chi-Square test was conducted using the best model results to understand the significance of explanatory variables (i.e., factors). All computational work was conducted in R. All code and processed data are available at this URL (https://github.com/kahot/SIV-R21 accessed on 9 December 2021).

We also examined the data (bam files) from Ita et al. [19] using the same methods described above to obtain the average diversity and divergence for comparison. This study described a cohort of four SIV_mac251_-infected rhesus macaques over 30 weeks. For calculating averages, the missing region (AA130–177) identified in our datasets was also excluded from the Ita et al. datasets.

### 2.7. Sequencing Quality Validation

To assess the error rate of this MiSeq assay, a portion of SIV_mac251_ *env* was amplified from the SIV challenge stock by RT-PCR. Production of cDNA was performed using the SuperScript III First-Strand Synthesis kit (Thermo Fisher) and PCR amplification was performed using Platinum PCR SuperMix High Fidelity (Thermo Fisher). The PCR product was TOPO TA cloned into the pCR 2.1 vector (Thermo Fisher) and transformed into *E. coli* (TOP10 cells, Thermo Fisher). A single bacterial plasmid clone was used for library preparation with the same conditions for PCR amplification steps as the cDNA made from the challenge stock and macaque viral RNA samples. The diversity of this control sample should be 0%, and therefore, the detected sequence diversity reflects the errors associated the library preparation and MiSeq sequencing. The average diversity detected in this control sample was 0.203%, with a median of 0.201% (ranged from 0 to 1.23%, Figure S1). Based on the results, the cutoff threshold for detecting a single mutation was set at 0.5% (for calculating the average diversity of a sample, all 585 sites were included).

## 3. Results

### 3.1. Diversity of the SIV Challenge Stock

**Figure 2:**
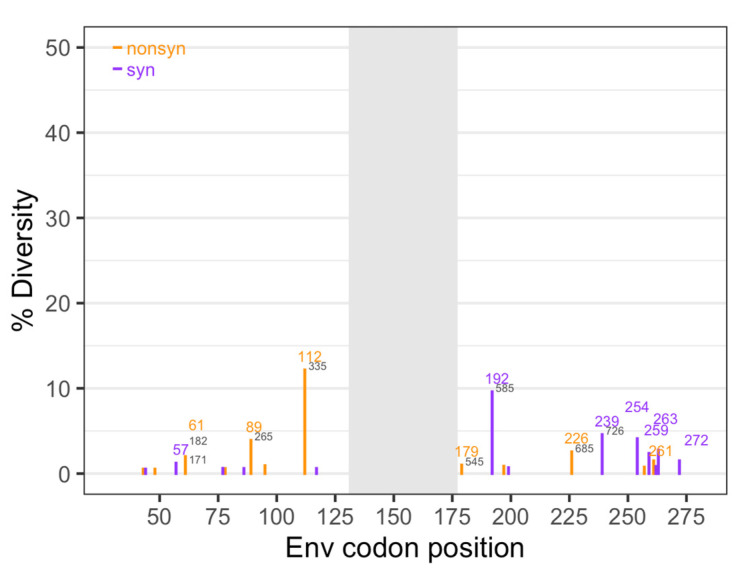
Diversity of the SIV challenge stock. Diversity was determined separately for nonsynonymous (orange) and synonymous (purple) changes at each nucleotide position, based on the challenge stock consensus sequence. Numbers in colors represent the amino acid position based on SIV_mac239_, and numbers in gray represent the nucleotide position based on SIV_mac251_. Gray shading indicates the region missing in some of the sequences.

All animals were infected with the same stock of SIV. To assess the diversity of the SIV challenge stock, we determined the consensus sequence for the challenge stock and called variants relative to the consensus nucleotide at each site. We calculated diversity over 588 sites from AA32 (nt94) to AA272 (nt825), not including AA130–177 because it was missing from many samples (indicated by a grey bar in Figure 2). Diversity was assessed separately for nonsynonymous and synonymous changes.

The average nonsynonymous diversity was 0.14% and the average synonymous diversity was slightly lower at 0.09%. Overall, diversity in the challenge stock was low, with a total diversity (synonymous and nonsynonymous combined) of 0.25% (Table 1). In comparison, the Ita study [19] also used SIV_mac251_, but the challenge stock was five times more diverse (1.54% for the same 588 sites) (Table 1, Figure S2). In the challenge stock used for this study, there were 25 nucleotide sites with diversity higher than our cutoff value of 0.5% (12 synonymous, and 13 nonsynonymous sites).

### 3.2. The SIV Transmitted/Founder (T/F) Variants

**Figure 3:**
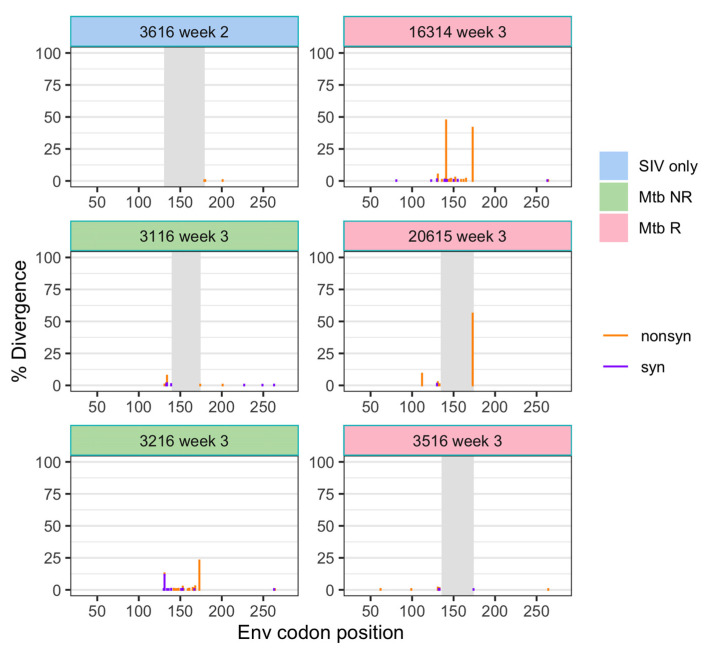
Divergence (from the stock consensus) of SIV env in plasma samples following transmission. Divergence is shown separately for nonsynonymous and synonymous changes at each position. Gray shading indicates the region missing in some of the sequences.

To understand the viral population bottleneck during transmission, we assessed the samples that were collected within 3 weeks after SIV infection, which included six animals from the three cohorts (Figure 3). Diversity in these early samples (0.21 ± 0.0005%) was slightly lower than in the stock virus (0.25%), which may indicate that there was a bottleneck at transmission. A nonsynonymous mutation at AA173 (nt527) was observed at a relatively high frequency (>20%) in three animals (3216, 16314, and 20615), as well as a nonsynonymous mutation at AA141 (nt428) in 16314 animal (Figure 3). However, these mutations fell into the missing region (AA130–177) of the challenge stock (see Materials and Methods), and therefore, we were unable to assess if they were present in the challenge stock or persisted over time.

### 3.3. SIV Diversity Mostly Stable over 6–9 Weeks

**Figure 4:**
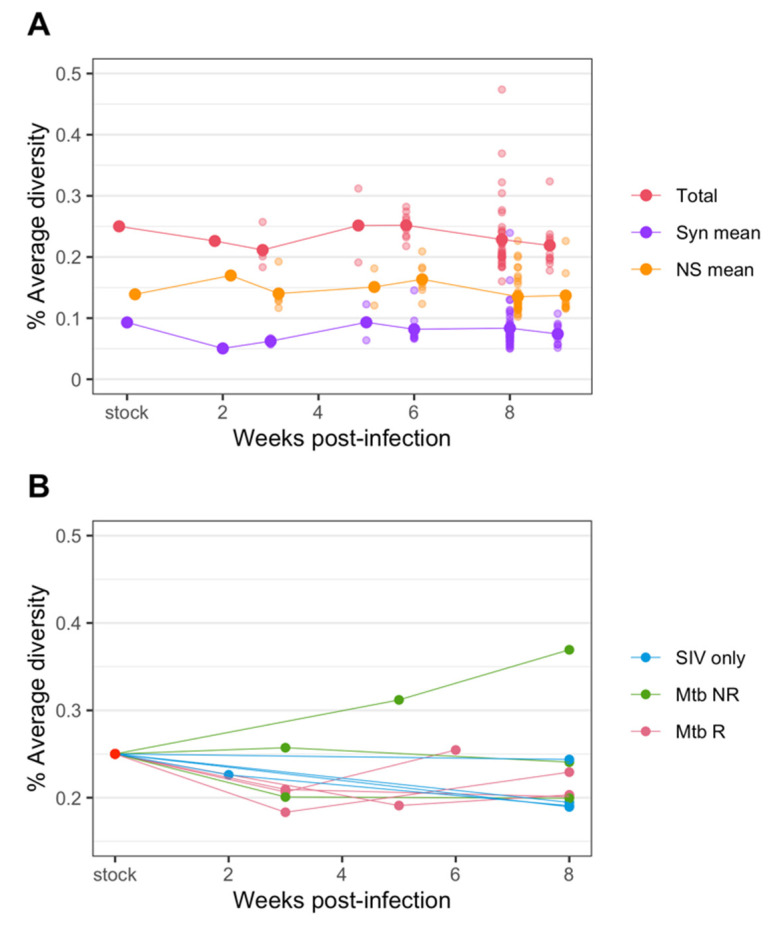
Changes in diversity of SIV env over the course of infection in plasma. (**A**) The average diversity of SIV of all samples at each time point. The total diversity (red) as well as diversity from synonymous (purple) and nonsynonymous changes (orange) are plotted. (**B**) The SIV average diversity of plasma samples only, colored by cohorts.

After the initial transmission bottleneck, the average diversity of all samples remained low and relatively constant over 6–9 weeks (Figure 4A). We also assessed diversity separately for synonymous and nonsynonymous mutations. The average nonsynonymous diversity was consistently higher than that of synonymous diversity (Figure 4A). This is consistent with results from Ita et al. [19], who also found that non-synonymous diversity was higher than synonymous diversity (Figure S3).

When we focus on viral diversity in plasma samples from individual animals, we also see that in most animals the diversity remained at a similar or slightly lower level compared to that of the challenge stock (Figure 4B). However, in some animals, diversity appeared to rebound after the initial transmission bottleneck. In one animal (Mtb NR, 30816) diversity ultimately reached a higher level than the challenge stock diversity. Generally, the viral diversity from the non-reactivator (Mtb NR) cohort showed the most variable responses, with one remaining the same level as the challenge stock, one decreased to around 0.2%, and the one increased above the stock diversity level as mentioned above. The viral diversity of the reactivator (Mtb R) cohort showed a distinct pattern from other cohorts; a decrease at post transmission but an increase at necropsy. The patterns of average viral diversity from the SIV only cohort was highly similar, trending down to below 0.2% at necropsy, except for one animal (3816) that remained at the same diversity level as the challenge stock.

### 3.4. SIV Diversity in Animal Cohorts and in Tissues

#### 3.4.1. Diversity Is Slightly Higher in the SIV/Mtb non-Reactivator Cohort

**Figure 5:**
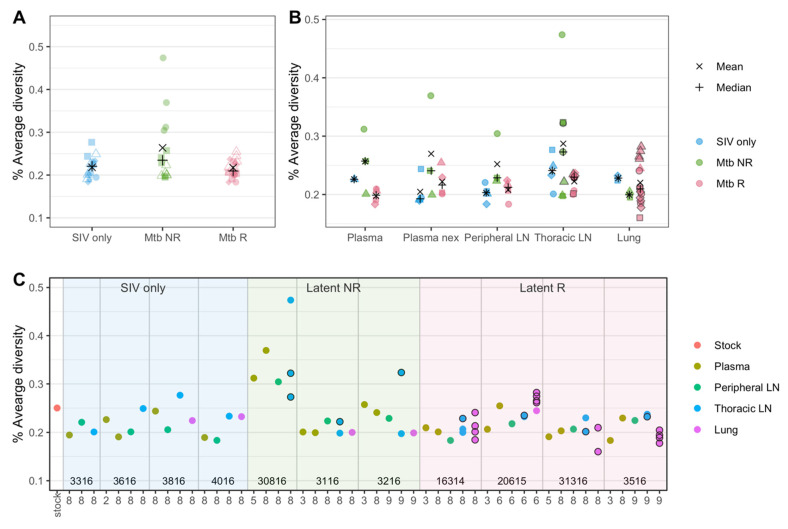
SIV nucleotide diversity in plasma and tissues. (**A**) Overall diversity of SIV in the three cohorts are shown. Here, all samples (all animals, all tissues and all time points) aggregated by cohort. Different shapes represent samples from different animals. (**B**) Diversity by each tissue type among the three cohorts are shown. Different shapes represent samples from different animals. (**C**) Genetic diversity of all available samples are shown, grouped by sample types in each animal. Animal numbers (listed in Table S1) are shown at the bottom and vertical lines distinguish animals. The *x*-axis represents the week post-infection from which each sample was obtained. Multiple samples were available for lungs and thoracic LNs in some animals and are shown in the same columns. (**B**,**C**) show the same samples as (**A**) (all animals, all tissues and all timepoints) but separatee by tissue type (**B**) and animal (**C**). Black bordered shapes in (**B**,**C**) represent samples in which a granuloma was identified at necropsy. ‘Plasma’ represent plasma samples at an early time point, and ‘Plasma nex’ represents plasma samples taken at necropsy.

We compared SIV diversity between the three cohorts in the plasma and in all tissue types. The comparison showed that, overall, the samples from the non-reactivator (Mtb NR) cohort had slightly higher average diversity than those from the other two cohorts, though none of the comparisons were statistically significant by Mann–Whitney test (adjusted *p*-values = 0.179 (Mtb NR vs. Mtb R), 0.217 (Mtb NR vs. SIV only), 0.99 (SIV only vs. Mtb R), Figure 5A). Comparing the average SIV diversity between the cohorts for different tissues followed a similar trend: Slightly higher average diversity in the non-reactivator (Mtb NR) cohort was observed in all tissue types, with the exception of lung tissue, in which the diversity level was reversed, and non-reactivator had the lowest average diversity (Figure 5B, none were statistically significant, Mann–Whitney test, adjusted p-values ranged 0.86–1). When focusing just on the tissues (regardless of treatment group), slightly higher average diversity was observed in thoracic LNs than others, though again, the results were not statistically significant (Mann–Whitney test, adjusted p-values ranged 0.66–1) (Figure 5B).

To overcome the low statistical power due to the limited sample size, we applied GLMM to further understand the differences in the levels of SIV diversity between cohorts and tissue types. The best fit model included sampling week, cohort, and tissue types as fixed effects and individual animal as a random effect. The model results confirmed our initial observations: Among the different tissue types, ‘Thoracic LN’ (*p*-value = 0.023) was identified as a significant coefficient, and among the cohort groups, ‘Mtb NR’ (*p*-value = 0.053) was identified as a marginally significant coefficient in the best model (Table S3), suggesting that the viral diversity level in thoracic LNs was significantly different (38% higher) and the diversity in the Mtb NR cohort was marginally significantly different (20% higher) from that of SIV-only plasma samples while others did not (Figure S4). The subsequent Wald chi-Square test, which assesses which explanatory variables in a model were significant, revealed that all three effects (sampling week, cohort, and tissue types) were marginally significant (Sampling week: *p*-value = 0.0785, Cohort: *p*-value = 0.0621, Tissues types: *p*-value = 0.0559).

Since higher viral replication is expected to lead to higher diversity, we assessed the SIV RNA copy numbers in each tissue sample to determine if viral replication affected the observed differences in SIV diversity between tissues. We found that in the lung samples, where the non-reactivator (Mtb NR) cohort had the lowest average SIV diversity, the RNA copy numbers followed a similar trend. Indeed, the non-reactivator cohort had a several orders of magnitude lower RNA copy numbers in lungs than other cohorts (Table 2). This could indicate that lower SIV replication in the lungs of co-infected animals without Mtb reactivation may have been associated with observed lower viral diversity. However, when compared across all samples, overall RNA copy numbers of tissues were not correlated to their average SIV diversities (Spearman’s rank correlation, *p*-value = 0.381).

We assessed whether SIV diversity differed between samples from the lung and LN with evidence of granuloma involvement at necropsy (Figure 5B,C, outlined circles). Overall, SIV diversity did not differ between granuloma and non-granuloma samples (Figure S5A, Mann–Whitney test, *p*-value = 0.65). Within granuloma samples, SIV diversity was significantly higher in the non-reactivator (Mtb NR) cohort than the reactivator (Mtb R) cohort (Mann–Whitney test, *p*-value = 0.026, Figure S5B), though we should note that the non-reactivator granulomas were all from thoracic LNs and the reactivator granulomas were mostly from lungs (Figure S5C). We also assessed the relationship between the SIV diversity and Mtb bacterial burden (as measured by colony forming units) in the samples, which showed no correlation (Spearman’s rank correlation, *p*-value = 0.366 (granulomas), *p*-value = 0.111 (non-granulomas)).

**Figure 6:**
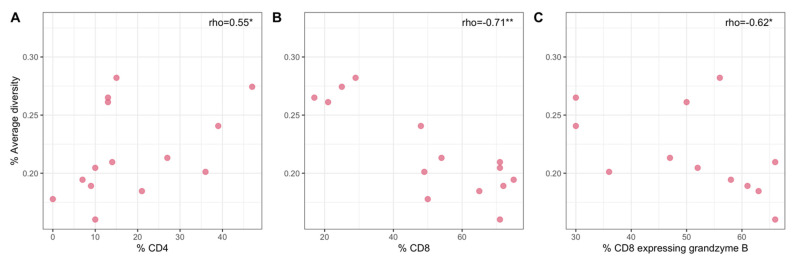
Relationships between SIV diversity and the frequency of CD4+ T cells (**A**), the frequency of CD8+ T cells (**B**), and the frequency of CD8+ T cells expressing granzyme B (**C**) in lung granuloma samples of the Mtb R cohort. Asterisks denote * for *p*-value < 0.05, ** for <0.01.

In a prior study we characterized immune responses within individual granulomas for the animals in this study [23] (Table S2). Within individual lung granulomas, viral diversity had significant positive correlation with the frequency of CD4+ T cells (Spearman’s rank correlation, *ρ* = 0.551, *p*-value = 0.041) (Figure 6A). In contrast, the frequency of CD8+ T cells was negatively correlated with viral diversity (Spearman’s rank correlation, *ρ* = −0.713, *p*-value = 0.0042) (Figure 6B). The CD4/CD8+ cell ratio consequently showed a positive correlation with viral diversity (Spearman’s rank correlation, *ρ* = 0.755, *p*-value = 0.00181). Granzyme B has an important role in CD8-mediated cytotoxity [31] and is known to be important in HIV and SIV control [32]. Accordingly, the frequency of CD8+ T cells expressing granzyme B was negatively correlated with viral diversity (Spearman’s rank correlation, *ρ* = −0.624, *p*-value = 0.030) (Figure 6C).

#### 3.4.2. Genetic Drift Similar between Cohorts and Tissues

Population bottlenecks can lead to drastic changes in mutation frequencies, which is referred to as genetic drift. We predicted that viruses might undergo a bottleneck during the infection of tissues from the blood. For this analysis, we included for each animal, the nucleotide positions that had relatively high mutation frequencies (>0.8%) at necropsy (weeks 6–9 post-SIV infection) in at least one sample. Therefore, nucleotide positions chosen were animal specific. We assessed the frequency differences of each mutation between the plasma and three tissue types (peripheral LN, thoracic LN, and lung) within each animal. There were 10 to 57 high frequency (>0.8%) nucleotide positions per animal. The average difference in frequencies (absolute differences averaged over all animals) between plasma and tissues was 3.13%. The average difference between plasma and peripheral LN was 2.30%, between plasma and thoracic LN was 3.74%, and between plasma and lung was 3.43%. These frequency differences among tissue types were not significantly different (Mann–Whitney test, adjusted *p*-value = 0.18–0.97).

**Figure 7:**
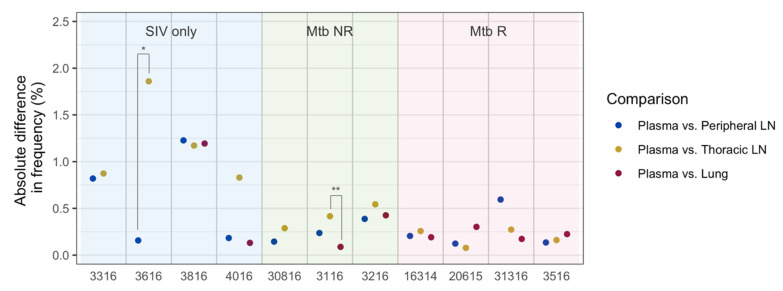
Differences in nucleotide mutation frequencies between plasma and tissues at necropsy in each animal. Frequency differences were calculated from the relatively high frequency sites (>0.8%) for the given animal. Each dot represents a median absolute difference in frequencies. Asterisks (*) indicate statistical significance by the Mann–Whitney test (* = an adjusted *p*-value < 0.05, ** = an adjusted *p*-value < 0.01).

We further looked at the effects of genetic drift within each animal, and determined if the effects of drift were different between tissues (Figure 7). Only two out of all pair-wise comparisons (*n* = 27) were found to be significantly different, both of which were between thoracic LN and another tissue type (Mann–Whitney test, adjusted *p*-value = 0.005 for Animal 3116, and 0.040 for Animal 3616, Figure 7), indicating that the viral population in thoracic LN may have experienced greater genetic drift than that in peripheral LN or lung.

Genetic drift is likely stronger in smaller populations. Therefore, we assessed the relationship between RNA copy numbers and mutation frequency differences between tissues. However, absolute differences in frequencies were not correlated with RNA copy numbers (Spearman’s rank correlation test, *p*-value = 0.63).

### 3.5. Amino Acid Substitutions

#### 3.5.1. Amino Acid R112K G→A Mutation Common in Two Monkeys

**Figure 8:**
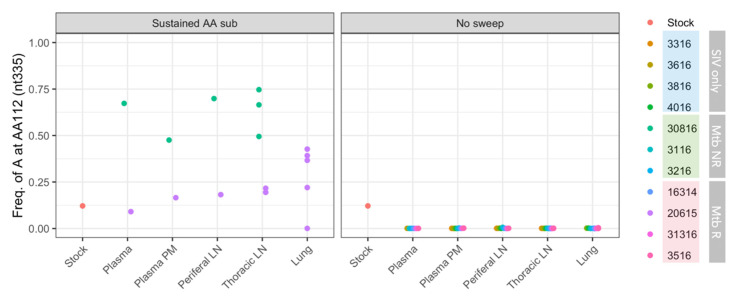
Frequencies of adenosine at nt335 (results in R112K substitution) in SIV isolated from different animals. Each dot represents a single sample. ‘Plasma’ represent plasma samples at an early time point, and ‘Plasma nex’ represents plasma samples at necropsy. Animal 30816 did not have lung samples.

The R112K substitution (nucleotide G→A mutation) was present in the SIV challenge stock at 12.1%. While the function of this mutation is unknown, we are reporting it here because of the surprising frequency changes in two animals (Figure 8). The mutation was undetectable in nine of the animals (82%) and did not become detectable in any samples in these animals (all observed frequencies below the cutoff threshold of 0.5%). In two co-infected animals, 20615 (Mtb R) and 30816 (Mtb NR), the mutation remained detectable. In 20615, the mutation was sustained at a frequency close to that of the SIV challenge stock. In 30816 on the other hand, the mutation became the majority in the viral population at week 5 post-infection, and was found at a high frequency across all tissue types at week 8 post-infection.

#### 3.5.2. Amino Acid Substitutions Associated with Neutralizing Antibody Escape Are Detected but Rare

Previous studies have reported Env amino acid substitutions involved in neutralizing antibody escape at nine positions present in our sequenced region (SIV_mac239_ AA 120, 132, 135,136,138,139,198,201, and 202; see Table 1 of Ita et al. [18]). We investigated whether substitutions occurred at the same positions in our samples. A total of 17 different types of substitutions at seven positions were observed at a frequency greater than 0.5% (Table S3). Of these, K120R, a substitution that occurs outside of V1 loop and reported in several previous studies [19,22,33], was observed with the highest average frequency (5.5%) in 9 of our samples (13%). Ita et al. [19] observed a rapid substitution of K120R immediately following initial infection (≤2 weeks) in one of four monkeys. This study also showed a *de novo* mutation resulting in K120R in another monkey at a later time point (>8 weeks). However, the observed frequencies in our samples were low with the highest at 19.8% in thoracic LN of one Mtb R animal (Table S4).

### 3.6. Indels

The Ita et al. study [19] reported the appearance of various indels near the V1 loop from an early stage of infection. However, very few indels were observed in samples from this study, as well as in the challenge stock virus (Figure S6); none of the indels had a frequency above our detection threshold (0.5%) in any samples. Compared to SIV_mac239_, the SIV_mac251_ reference genome (MK686248) and our stock consensus sequence had two insertions. They were a 6-nucleotide insertion between amino acid positions 126 and 127 (SIV_mac239_ positioning) and a 3-nucleotide insertion between amino acid positions 141 and 142. These two insertions were consistently present in all of our samples (i.e., no sequences were missing these nine bases). No further analysis on indels were, therefore, performed in this study.

## 4. Discussion

HIV-TB co-infection in humans is associated with increased HIV-1 replication and diversity, though the mechanism is poorly understood (reviewed in [34]). Our study assessed genetic diversity of SIV in macaques infected with SIV only, or co-infected with Mtb. The number of studies on HIV/SIV and Mtb co-infection have increased in recent decades, but most of these studies have focused on clinical, immunological, or pharmacological aspects [35,36,37,38,39,40,41], and little is known about how co-infection influences the viral evolutionary dynamics in hosts. This study was the first to assess in vivo SIV diversity not only in the blood, but also the lungs and LNs (peripheral and thoracic), including Mtb granulomas. While the number of animals in each of the three cohorts (Mtb reactivator animals, Mtb non-reactivator animals, SIV only animals) was small and viral diversity was low, this study gives a first look into the dynamics of SIV in the tissues of SIV/Mtb co-infected animals. Our study results therefore contribute to our understanding of host-pathogen interactions during SIV/HIV co-infection and its pathogenesis.

Overall, SIV/Mtb co-infection had little effect on viral diversity during the study period of initial SIV infection: We did not observe any large differences in viral diversity among cohorts or between tissue types. This may have been due to (1) low diversity of the SIV_mac251_ challenge stock used in this study, resulting in the transmission of an almost clonal virus population, and/or (2) relatively low in vivo SIV_mac251_ replication during acute infection due to the limited pathogenicity in cynomolgus macaques compared to other macaque SIV models. However, previous studies of SIV infection have also reported low viral diversity in plasma during the first few months of infection, with nucleotide diversity typically less than 1%. For example, Ita et al. [19] investigated evolutionary dynamics of SIV_mac251_ in rhesus macaques, in which infection was more pathogenic, but the diversity of SIV *env* did not increase until 14 weeks post-infection and it was not until 23 weeks post-infection that the diversity reached the level of the challenge stock (~1.5%). Similarly, in the Burns and Desrosiers study [18] of SIV *env* in rhesus macaques infected with SIV_mac239_, few nucleotide (0.4%) and amino acid (0.7%) substitutions were observed initially (6 weeks post-infection), which increased more than 5-fold at 69 weeks post-infection. These data suggest that the short duration of SIV infection likely had a greater influence on the limited SIV diversity in this study than the non-pathogenic nature of virus in cynomolgus macaques. In addition, we believe that using a more diverse virus stock, and ideally a more pathogenic virus, would help detect selection on specific mutations and divergence between tissue compartments through transmission of more variants and more robust viral replication.

Despite the low overall viral diversity, we observed a small transmission bottleneck of SIV, resulting in 0.04% reduction in the total average diversity in early plasma samples relative to that of the challenge stock, consistent with previous observations in SIV and HIV transmission studies [19,42,43,44]. A bottleneck can also occur when virus infection disseminates between plasma and tissues within an individual [44,45]. A small change in allele frequencies was observed between plasma and tissues (average 3.1% for sites with frequencies >0.8%), which suggests that the viral populations experienced genetic drift between the blood and tissues possibly due to compartmentalization of replication or differences in target cell populations. Certainly the quantity and quality of antigen-specific T cells differ significantly between compartments, as well as among individual granulomas within the same animal [46]. This may lead to differences in viral replication, resulting in divergence of viral populations between tissues.

Although the diversity differences observed between SIV only and SIV/Mtb co-infected animals were small, some interplay between SIV and Mtb appeared in this study. We previously reported that SIV infection of Mtb-infected animals resulted in greater rates of TB reactivation, and co-infection was associated with more disease progression and greater bacterial burden [23,46]. Also, SIV RNA copy numbers were greater in individual lung granulomas of reactivator animals (Mtb R) than in non-reactivator animals (Mtb NR) [23]. These findings led us to expect viral diversity to be higher in reactivator animals. Instead, we found that (1) overall RNA copy numbers did not correlate with viral diversity, (2) overall average viral diversity appeared to be slightly higher (20%) in non-reactivator animals than reactivator animals (Figure S4B), and (3) no differences in viral diversity existed between granuloma and non-granuloma samples in this study. When we focused on the lung samples, we observed that the lungs of non-reactivator animals had the lowest RNA copy numbers and the lowest average viral diversity, which could suggest a possibility that reactivation of Mtb is associated with higher SIV replication in the lung. However, these results need to be interpreted carefully, as our study is based on a small number of samples with low viral diversity with limited statistical power. Further studies are needed to confirm these results with more certainty.

Heterogeneity of immune responses between individual granulomas is known to occur [16], which could mean that some granulomas allow more SIV replication than others. Consistent with these data, we observed a negative correlation between viral diversity and frequency of CD8+ T cells and a positive correlation between viral diversity and frequency of CD4+ T cells from lung granulomas of reactivator animals (no lung granulomas were available for non-reactivator animals). Thus, it is not surprising that the CD4/CD8 ratio is also positively correlated with viral diversity. CD8+ T cells play a key role in reducing HIV viral replication, as several mechanisms of CD8+ T cell driven control of HIV infection have been described during both acute and chronic infection [47]. As replication is required for viral genetic diversity, it is reasonable to assume that higher CD8+ T cell responses and lower viral replication during acute SIV infection would be associated with lower viral diversity. Moreover, we observed that the frequency of granzyme B-expressing CD8+ T cells in the granulomas was also negatively correlated with viral diversity, as granzyme B is a known mediator of cytotoxic function in HIV and SIV infection [32]. In contrast, the relationship between CD4+ T cell count and HIV genetic diversity appears to be variable [46]. Though not addressed in this study, it is worth mentioning macrophages (e.g., alveolar macrophages) can be infected by both HIV and SIV [48,49] as well as Mtb, particularly in the granuloma. We speculate that macrophages interaction with T cells in the granuloma may also contribute to viral replication and diversity. It is difficult to extrapolate our findings to the literature, as our data are limited to early time points after SIV infection and to the unique microenvironment of individual lung granulomas during Mtb infection. To our knowledge, correlations with SIV viral dynamics in the context of this experimental model have not been described and show potentially unique interactions within the granuloma during Mtb-SIV co-infection.

Interestingly, SIV diversity was highest in thoracic LNs compared to the plasma and other tissues. In addition, there was evidence from our genetic drift analysis that the virus from the thoracic LNs was somewhat differentiated from the virus in other tissues. Thoracic LNs are the primary sites of T cell trafficking from their regional lung lobes, where the majority of granulomas are present, and likely differ in their immunologic environments as a reflection of the heterogeneous nature of TB disease (i.e., disease may involve only two of seven lung lobes). Moreover, the thoracic LN can be directedly infected with Mtb, leading to remodeling of the lymph node architecture with granuloma formation. It is a unique immunologic environment as underscored by the fact that poor Mtb killing is observed compared to lung granulomas [50]. This environment appears to lead to greater SIV replication and may be important for dissemination of Mtb during co-infection, leading to greater incidence of extrapulmonary disease. Future studies of SIV dynamics in the thoracic LNs are warranted.

The main limitation of our study was the low viral diversity and the small number of animals with a limited number of samples for each tissue type due to technical difficulties. This made some comparisons hard and others impossible (e.g., we had no lung granuloma samples from non-reactivators). The use of both genetically barcoded Mtb [24] and barcoded SIV [51] during co-infection may allow better tracking of both pathogens within the animals during co-infection. In addition, a more diverse virus stock and longer SIV infection will make it possible to observe selection on individual mutations in cohorts, animals, or specific tissues. The lungs and thoracic LNs, especially those containing granulomas, should be carefully examined in subsequent studies for virus and bacterial replication and immunopathogenesis.

Co-infection with Mtb and HIV is a worldwide public health concern, leading to worse clinical outcomes caused by both pathogens, especially in areas with decreased access to antibiotic and antiretroviral treatments. As both SIV and Mtb infect immune cells present in the same tissues, their replication is exacerbated by unchecked or dysregulated immune responses, making co-infection complicated to study. A better understanding of the evolutionary dynamics of both pathogens is needed to effectively prevent and treat both infections. While increasing numbers of studies are underway to evaluate the intricate dynamics of HIV/Mtb co-infection, they focus primarily on immunological and clinical responses. Research on viral evolutionary responses during co-infection are lagging. Our study results support the usefulness and validity of the SIV/Mtb macaque model to assess viral evolutionary dynamics by illuminating the interplay of both pathogens in vivo.

## Figures and Tables

**Table 1:** Comparison of diversity of the SIV challenge stocks used in this study (SIV/Mtb) and in the Ita et al. [19] study.

	Total	Synonymous	Nonsynonymous
SIV/Mtb	0.250	0.093	0.139
Ita et al. study [18]	1.544	0.418	1.075

**Table 2:** Median RNA copy numbers (RNA copies × 10^6^/g tissue (lower–upper range)) are shown for each tissue type from the three cohorts. *n* = the number of samples.

	Lung	Peripheral LN	Thoracic LN
SIV only	13.02 (0.82–25.2) (*n* = 2)	19.73 (1.56–92.1) (*n* = 4)	166.1 (19.2–450.4) (*n* = 4)
Mtb NR	0.029 (0.013–0.045) (*n* = 2)	28.32 (12.3–129.4) (*n* = 3)	14.68 (2.09–644.6) (*n* = 7)
Mtb R	0.39 (0.028–16.1) (*n* = 15)	19.76 (3.76–209.1) (*n* = 4)	17.85 (0.16–89.0) (*n* = 9)

## Data Availability

Processed bam files of SIV from each animal are available at https://doi.org/10.6084/m9.figshare.14478273 (accessed on 9 December 2021). All other relevant data are within the manuscript and its Supporting Information files and at the GitHub repository (https://github.com/kahot/SIV-R21 (accessed on 9 December 2021)).

## Supplementary Materials

The following are available online at https://www.mdpi.com/article/10.3390/v14010048/s1, Figure S1: Diversity of a cDNA clone of SIV_mac251_ *env,* Figure S2: Diversity of the challenge virus (SIV_mac251_) from this study (SIV-Mtb) and the Ita et al. study, Figure S3: Changes in diversity of SIV *env* over the course of infection from this study and the Ita et al. study, Figure S4: The estimated SIV diversity by the GLMM (blue, round) and the observed average diversity (red, diamond), Figure S5: SIV diversity in granuloma vs. non-granuloma tissues. Figure S6: Observed insertion and deletion mutations in the SIV_mac251_ challenge stock used in this study, Table S1: Animal IDs and sample information used in this study, Table S2: SIV Viral RNA copy numbers and CD4+ T-cell information for samples used in this study, Table S3: Results from the generalized linear mixed model on SIV diversity, Table S4: SIV *env* substitutions observed in this study that were previously reported.

## References

[1] (2019). UNAIDS Tuberculosis and HIV: Progress towards the 2020 Target. https://www.unaids.org/en/resources/documents/2019/tuberculosis-and-hiv-progress-towards-the-2020-target.

[2] Waters R., Ndengane M., Abrahams M.-R., Diedrich C.R., Wilkinson R.J., Coussens A.K. (2020). The Mtb-HIV Syndemic Interaction: Why Treating M. Tuberculosis Infection May Be Crucial for HIV-1 Eradication. Future Virol..

[3] Goletti D., Weissman D., Jackson R.W., Graham N.M., Vlahov D., Klein R.S., Munsiff S.S., Ortona L., Cauda R., Fauci A.S. (1996). Effect of Mycobacterium Tuberculosis on HIV Replication. Role of Immune Activation. J. Immunol..

[4] Biru T., Lennemann T., Stürmer M., Stephan C., Nisius G., Cinatl J., Staszewski S., Gürtler L.G. (2010). Human Immunodeficiency Virus Type-1 Group M Quasispecies Evolution: Diversity and Divergence in Patients Co-Infected with Active Tuberculosis. Med. Microbiol. Immunol..

[5] Collins K.R., Mayanja-Kizza H., Sullivan B.A., Quiñones-Mateu M.E., Toossi Z., Arts E.J. (2000). Greater Diversity of HIV-1 Quasispecies in HIV-Infected Individuals with Active Tuberculosis. J. Acquir. Immune. Defic. Syndr..

[6] Abrahams M.R., Anderson J.A., Giorgi E.E., Seoighe C., Mlisana K., Ping L.H., Athreya G.S., Treurnicht F.K., Keele B.F., Wood N. (2009). Quantitating the Multiplicity of Infection with Human Immunodeficiency Virus Type 1 Subtype C Reveals a Non-Poisson Distribution of Transmitted Variants. J. Virol..

[7] Gottlieb G.S., Nickle D.C., Jensen M.A., Wong K.G., Grobler J., Li F., Liu S.-L., Rademeyer C., Learn G.H., Karim S.S.A. (2004). Dual HIV-1 Infection Associated with Rapid Disease Progression. Lancet.

[8] Grobler J., Gray C.M., Rademeyer C., Seoighe C., Ramjee G., Karim S.A., Morris L., Williamson C. (2004). Incidence of HIV-1 Dual Infection and Its Association with Increased Viral Load Set Point in a Cohort of HIV-1 Subtype C-Infected Female Sex Workers. J. Infect. Dis..

[9] Sagar M., Lavreys L., Baeten J.M., Richardson B.A., Mandaliya K., Chohan B.H., Kreiss J.K., Overbaugh J. (2003). Infection with Multiple Human Immunodeficiency Virus Type 1 Variants Is Associated with Faster Disease Progression. J. Virol..

[10] Whalen C., Horsburgh C.R., Hom D., Lahart C., Simberkoff M., Ellner J. (1995). Accelerated Course of Human Immunodeficiency Virus Infection after Tuberculosis. Am. J. Respir. Crit. Care. Med..

[11] WHO (2019). Global Tuberculosis Report (WHO/CDS/TB/2019.15). https://apps.who.int/iris/rest/bitstreams/1257851/retrieve.

[12] Evans D.T., Silvestri G. (2013). Non-Human Primate Models in AIDS Research. Curr. Opin. HIV AIDS.

[13] Flynn J.L., Gideon H.P., Mattila J.T., Lin P.L. (2015). Immunology Studies in Non-Human Primate Models of Tuberculosis. Immunol. Rev..

[14] Feder A.F., Kline C., Polacino P., Cottrell M., Kashuba A.D.M., Keele B.F., Hu S.-L., Petrov D.A., Pennings P.S., Ambrose Z. (2017). A Spatio-Temporal Assessment of Simian/Human Immunodeficiency Virus (SHIV) Evolution Reveals a Highly Dynamic Process within the Host. PLoS Pathog..

[15] Feder A.F., Pennings P.S., Hermisson J., Petrov D.A. (2019). Evolutionary Dynamics in Structured Populations under Strong Population Genetic Forces. G3: Genes Genomes Genet..

[16] Gideon H.P., Phuah J., Myers A.J., Bryson B.D., Rodgers M.A., Coleman M.T., Maiello P., Rutledge T., Marino S., Fortune S.M. (2015). Variability in Tuberculosis Granuloma T Cell Responses Exists, but a Balance of pro- and Anti-Inflammatory Cytokines Is Associated with Sterilization. PLoS Pathog..

[17] Danaviah S., de Oliveira T., Gordon M., Govender S., Chelule P., Pillay S., Naicker T., Cassol S., Ndung’u T. (2016). Analysis of Dominant HIV Quasispecies Suggests Independent Viral Evolution Within Spinal Granulomas Coinfected with Mycobacterium Tuberculosis and HIV-1 Subtype C. AIDS Res. Hum. Retrovir..

[18] Burns D.P., Desrosiers R.C. (1991). Selection of Genetic Variants of Simian Immunodeficiency Virus in Persistently Infected Rhesus Monkeys. J. Virol..

[19] Ita S., Hill A.K., Lam E.C., Dufort F.J., Yang X., Newman R., Leviyang S., Fofana I.B., Johnson W.E. (2018). High-Resolution Sequencing of Viral Populations During Early SIV Infection Reveals Evolutionary Strategies for Rapid Escape from Emerging Env-Specific Antibody Responses. J. Virol..

[20] Sato S., Yuste E., Lauer W.A., Chang E.H., Morgan J.S., Bixby J.G., Lifson J.D., Desrosiers R.C., Johnson W.E. (2008). Potent Antibody-Mediated Neutralization and Evolution of Antigenic Escape Variants of Simian Immunodeficiency Virus Strain SIVmac239 In Vivo. J. Virol..

[21] Rybarczyk B.J., Montefiori D., Johnson P.R., West A., Johnston R.E., Swanstrom R. (2004). Correlation between Env V1/V2 Region Diversification and Neutralizing Antibodies during Primary Infection by Simian Immunodeficiency Virus Sm in Rhesus Macaques. J. Virol..

[22] Overbaugh J., Rudensey L.M., Papenhausen M.D., Benveniste R.E., Morton W.R. (1991). Variation in Simian Immunodeficiency Virus Env Is Confined to V1 and V4 during Progression to Simian AIDS. J. Virol..

[23] Diedrich C.R., Rutledge T., Maiello P., Baranowski T.M., White A.G., Borish H.J., Karell P., Hopkins F., Brown J., Fortune S.M. (2020). SIV and Mycobacterium Tuberculosis Synergy within the Granuloma Accelerates the Reactivation Pattern of Latent Tuberculosis. PLoS Pathog..

[24] Martin C.J., Cadeba A.M., Leung V.W., Lin P.L., Maiello P., Hicks N., Chase M.R., Flynn J.L., Fortune S.M. (2017). Digitally Barcoding Mycobacterium Tuberculosis Reveals in Vivo Infection Dynamics in the Macaque Model of Tuberculosis. mBio.

[25] Zhou S., Bednar M.M., Sturdevant C.B., Hauser B.M., Swanstrom R. (2016). Deep Sequencing of the HIV-1 Env Gene Reveals Discrete X4 Lineages and Linkage Disequilibrium between X4 and R5 Viruses in the V1/V2 and V3 Variable Regions. J. Virol..

[26] Bushnell B. (2018). BBTools. https://jgi.doe.gov/data-and-tools/bbtools/.

[27] Li H., Durbin R. (2009). Fast and Accurate Short Read Alignment with Burrows-Wheeler Transform. Bioinformatics.

[28] Li H. (2011). A Statistical Framework for SNP Calling, Mutation Discovery, Association Mapping and Population Genetical Parameter Estimation from Sequencing Data. Bioinformatics.

[29] Morgan M., Pagès H., Obenchain V., Hayden N. (2021). Rsamtools: Binary Alignment (BAM), FASTA, Variant Call (BCF), and Tabix File Import. https://bioconductor.org/packages/Rsamtools.

[30] R Core Team (2021). R Core Team R: A Language and Environment for Statistical Computing.

[31] Schneeweiss A., Wynne R.D., Marmor A. (1989). Effect of Flosequinan in Patients with Acute-Onset Heart Failure Complicating Acute Myocardial Infarction. Crit. Care Med..

[32] Roberts E.R., Carnathan D.G., Li H., Shaw G.M., Silvestri G., Betts M.R. (2016). Collapse of Cytolytic Potential in SIV-Specific CD8+ T Cells Following Acute SIV Infection in Rhesus Macaques. PLoS Pathog..

[33] Buckley K.A., Li P.-L., Khimani A.H., Hofmann-Lehmann R., Liska V., Anderson D.C., McClure H.M., Ruprecht R.M. (2003). Convergent Evolution of SIV Env after Independent Inoculation of Rhesus Macaques with Infectious Proviral DNA. Virology.

[34] Collins K.R., Quiñones-Mateu M.E., Toossi Z., Arts E.J. (2002). Impact of Tuberculosis on HIV-1 Replication, Diversity, and Disease Progression. AIDS Rev..

[35] Ahmed A., Rakshit S., Vyakarnam A. (2016). HIV-TB Co-Infection: Mechanisms That Drive Reactivation of *Mycobacterium Tuberculosis* in HIV Infection. Oral Dis..

[36] Bucşan A.N., Chatterjee A., Singh D.K., Foreman T.W., Lee T.-H., Threeton B., Kirkpatrick M.G., Ahmed M., Golden N., Alvarez X. (2019). Mechanisms of Reactivation of Latent Tuberculosis Infection Due to SIV Coinfection. J. Clin. Investig..

[37] Ellis A.L., Balgeman A.J., Larson E.C., Rodgers M.A., Ameel C., Baranowski T., Kannal N., Maiello P., Juno J.A., Scanga C.A. (2020). MAIT Cells Are Functionally Impaired in a Mauritian Cynomolgus Macaque Model of SIV and Mtb Co-Infection. PLoS Pathog..

[38] Foreman T.W., Mehra S., LoBato D.N., Malek A., Alvarez X., Golden N.A., Bucşan A.N., Didier P.J., Doyle-Meyers L.A., Russell-Lodrigue K.E. (2016). CD4^+^ T-Cell–Independent Mechanisms Suppress Reactivation of Latent Tuberculosis in a Macaque Model of HIV Coinfection. Proc. Natl. Acad. Sci. USA.

[39] Mattila J.T., Diedrich C.R., Lin P.L., Phuah J., Flynn J.L. (2011). Simian Immunodeficiency Virus-Induced Changes in T Cell Cytokine Responses in Cynomolgus Macaques with Latent *Mycobacterium Tuberculosis* Infection Are Associated with Timing of Reactivation. J. Immunol..

[40] Mehra S., Golden N.A., Dutta N.K., Midkiff C.C., Alvarez X., Doyle L.A., Asher M., Russell-Lodrigue K., Monjure C., Roy C.J. (2011). Reactivation of Latent Tuberculosis in Rhesus Macaques by Coinfection with Simian Immunodeficiency Virus: Reactivation of Latent TB. J. Med. Primatol..

[41] Wong K., Nguyen J., Blair L., Banjanin M., Grewal B., Bowman S., Boyd H., Gerstner G., Cho H.J., Panfilov D. (2020). Pathogenesis of Human Immunodeficiency Virus-Mycobacterium Tuberculosis Co-Infection. JCM.

[42] Keele B.F., Giorgi E.E., Salazar-Gonzalez J.F., Decker J.M., Pham K.T., Salazar M.G., Sun C., Grayson T., Wang S., Li H. (2008). Identification and Characterization of Transmitted and Early Founder Virus Envelopes in Primary HIV-1 Infection. Proc. Natl. Acad. Sci. USA.

[43] Leviyang S., Griva I., Ita S., Johnson W.E. (2017). A Penalized Regression Approach to Haplotype Reconstruction of Viral Populations Arising in Early HIV/SIV Infection. Bioinformatics.

[44] Ronen K., Sharma A., Overbaugh J. (2015). HIV Transmission Biology: Translation for HIV Prevention. AIDS.

[45] Joseph S.B., Swanstrom R., Kashuba A.D.M., Cohen M.S. (2015). Bottlenecks in HIV-1 Transmission: Insights from the Study of Founder Viruses. Nat. Rev. Microbiol..

[46] Diedrich C.R., Mattila J.T., Klein E., Janssen C., Phuah J., Sturgeon T.J., Montelaro R.C., Lin P.L., Flynn J.L. (2010). Reactivation of Latent Tuberculosis in Cynomolgus Macaques Infected with SIV Is Associated with Early Peripheral T Cell Depletion and Not Virus Load. PLoS ONE.

[47] McBrien J.B., Kumar N.A., Silvestri G. (2018). Mechanisms of CD8+ T Cell-Mediated Suppression of HIV/SIV Replication. Eur. J. Immunol..

[48] DiNapoli S.R., Ortiz A.M., Wu F., Matsuda K., Twigg H.L., Hirsch V.M., Knox K., Brenchley J.M. (2017). Tissue-Resident Macrophages Can Contain Replication-Competent Virus in Antiretroviral-Naive, SIV-Infected Asian Macaques. JCI Insight.

[49] Schiff A.E., Linder A.H., Luhembo S.N., Banning S., Deymier M.J., Diefenbach T.J., Dickey A.K., Tsibris A.M., Balazs A.B., Cho J.L. (2021). T Cell-Tropic HIV Efficiently Infects Alveolar Macrophages through Contact with Infected CD4+ T Cells. Sci. Rep..

[50] Ganchua S.K.C., White A.G., Klein E.C., Flynn J.L. (2020). Lymph Nodes—The Neglected Battlefield in Tuberculosis. PLOS Pathog..

[51] Fennessey C.M., Pinkevych M., Immonen T.T., Reynaldi A., Venturi V., Nadella P., Reid C., Newman L., Lipkey L., Oswald K. (2017). Genetically-Barcoded SIV Facilitates Enumeration of Rebound Variants and Estimation of Reactivation Rates in Nonhuman Primates Following Interruption of Suppressive Antiretroviral Therapy. PLOS Pathog..

