# Supplementary material for: SIV Evolutionary Dynamics in Cynomolgus Macaques during SIV-Mycobacterium tuberculosis Co-Infection
Source: Viruses. 2021 Dec 29;14(1):48. doi: 10.3390/v14010048 (PMC8778162; doi:10.3390/v14010048)
Supplement: Supplementary file 1 [file viruses-14-00048-s001.zip › viruses-1444073-Supplementary.pdf]

Supporting tables and figures for:

SIV evolutionary dynamics in cynomolgus macaques during SIV-*Mycobacterium tuberculosis* co-infection

Kaho H. Tisthammer, Christopher Kline, Tara Rutledge, Collin R. Diedrich, Sergio Ita, Philana Ling Lin, Zandrea Ambrose, Pleuni S. Pennings

Supplemental Figures

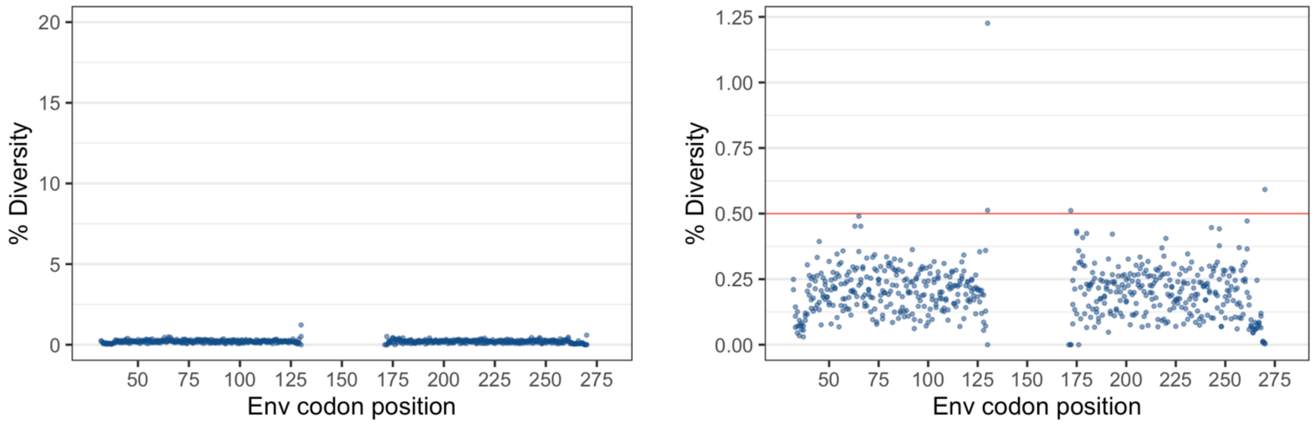

Figure S1. Diversity of a cDNA clone of SIV$_{mac251}$ *env*.

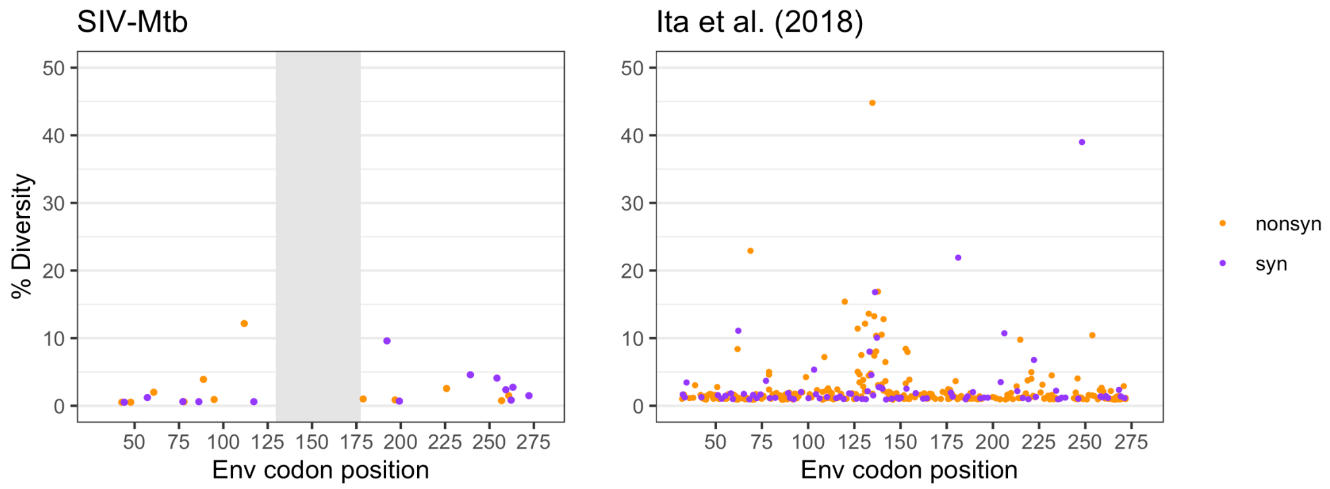

Figure S2. Diversity of the challenge virus (SIV_{mac251}) from this study (SIV-Mtb) and the Ita et al. study [1]. The gray section represents the region with no/low read counts.

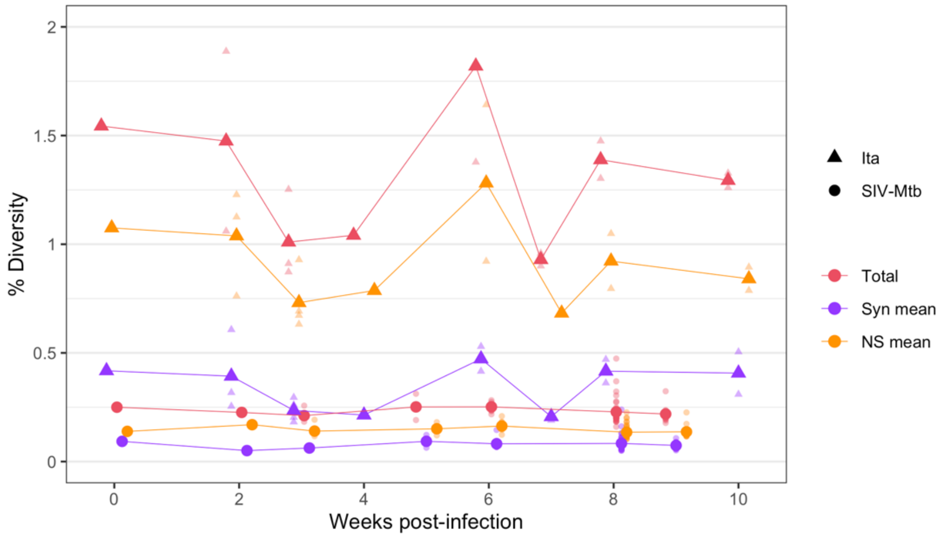

Figure S3. Changes in diversity of SIV *env* over the course of infection from this study and the Ita et al. study [1].

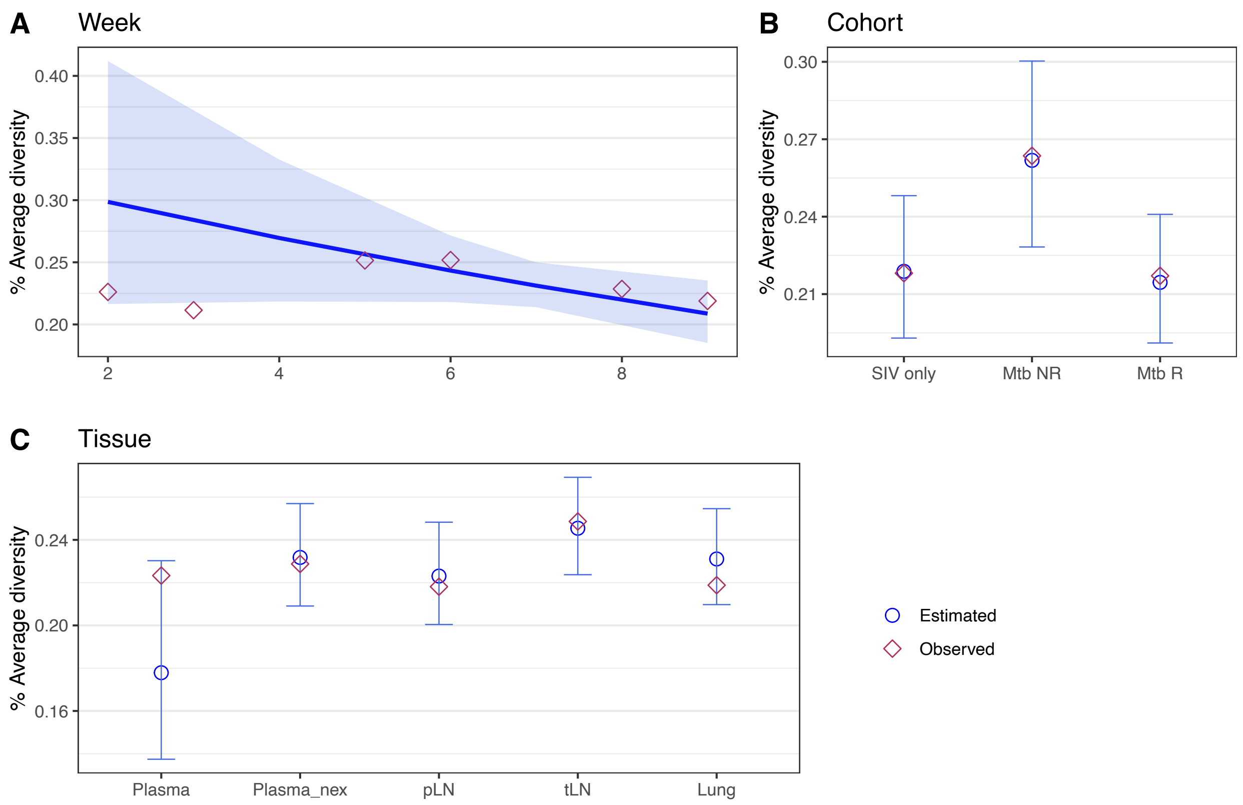

Figure S4. **The estimated SIV diversity by the GLMM (blue, round) and the observed average diversity (red, diamond).** The shaded area in A and error bars in B and C represent 95% confidence interval.

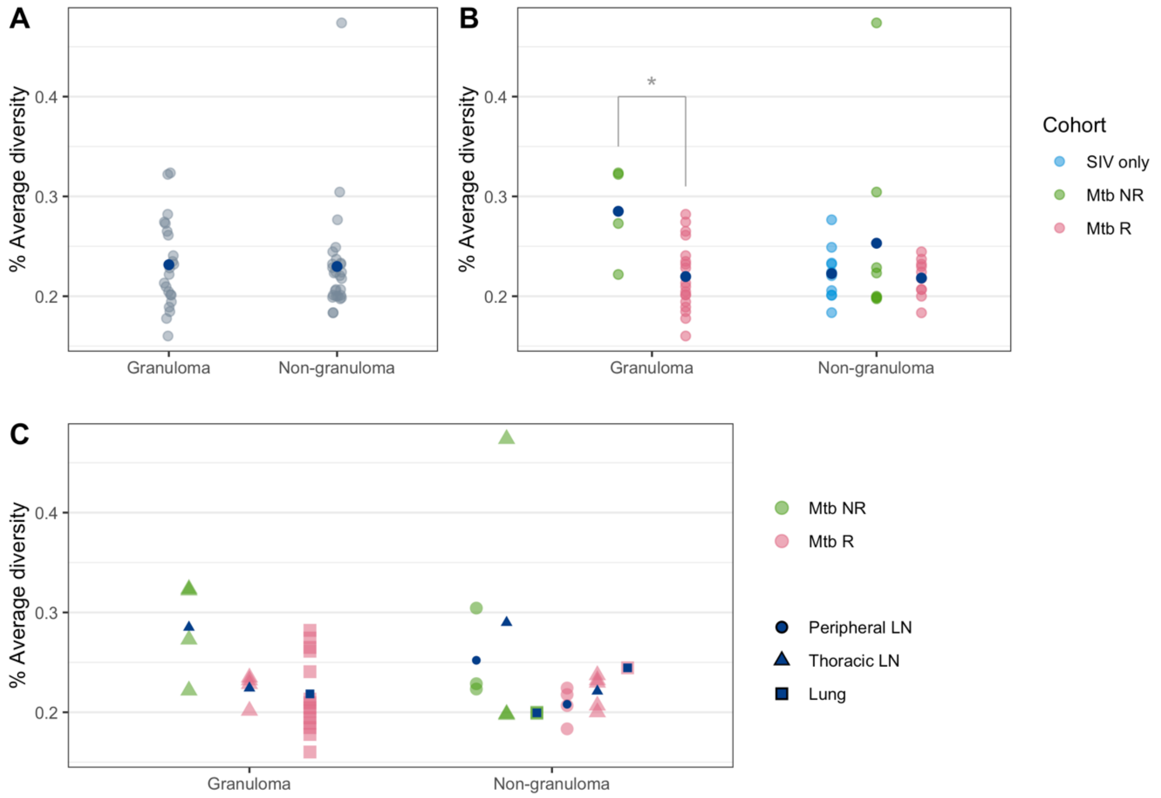

Figure S5. **SIV diversity in granuloma vs. non-granuloma tissues.** A) Overall comparison by pooling different tissues types, B) diversity in granuloma and non-granuloma samples by cohorts, and C) diversity in granuloma (n [number of animal] = 3 for Mtb NR, n = 4 for Thoracic Mtb R, and n = 4 for Lung LN Mtb R) and non-granuloma samples (n = 3 for Thoracic LN Mtb NR, n = 2 for Lung Mtb NR, n = 2 for Thoracic LN Mtb R, and n = 1 for Lung Mtb R) from different tissues of co-infected cohorts. Dark symbols represent averages. * indicates a statistically significant difference by Mann-Whitney test (*P*-value < 0.05).

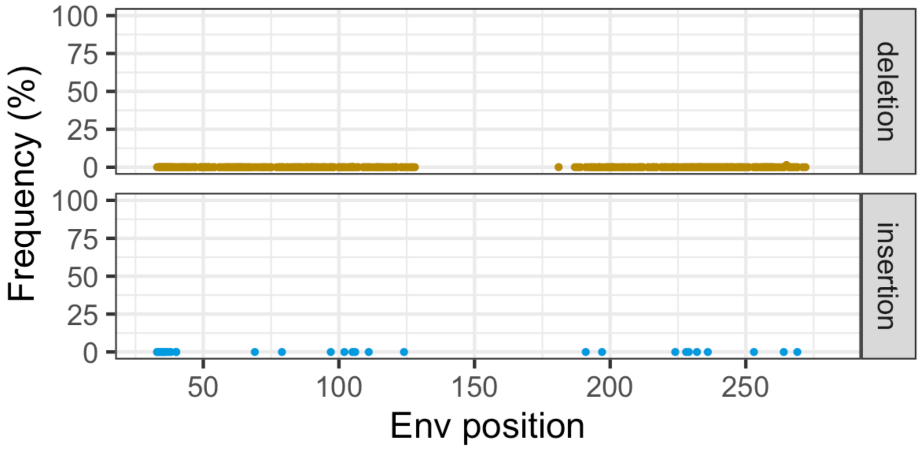

Figure S6. Observed insertion and deletion mutations in the SIV_{mac251} challenge stock used in this study.

Supplemental Tables

Table S1. **Animal IDs and sample information used in this study. All tissue samples were collected at necropsy.** The letter and the number below each Animal ID represent the sex (M = male) and the age of the animal.

L = left, R = right, C = central, LN= lymph node, HLN = hilar lymph node, cran = cranial, carin = carinal, ing = inguinal, LMS = left main stem, br = bronchus, **gran** = granuloma. For lungs: UL = upper lobe, LL = lower lobe, ML = middle lobe.

Cohort	Animal ID	Samples collected	Weeks of sample collection
SIV only	3316	Plasma	8
	M [6]	Peripheral LN (L ing), Thoracic LN (L cran HNL)	8
SIV only	3616	Plasma	2, 8
	M [6]	Peripheral LN (R ing), Thoracic LN (L cran HNL)	8
SIV only	3816	Plasma	8
	M [6]	Peripheral LN (L ing), Thoracic LN (L cran HNL), Lung (RLL)	8
SIV only	4016	Plasma	8
	M [7]	Peripheral LN (L ing), Thoracic LN (L cran HNL), Lung (LUL)	8
Mtb NR	30816	Plasma	5, 8
	M [5]	Peripheral LN (R ing), Thoracic LN [3] (L cran HLN, L cran HLN **gran**, LMS Br LN **gran**)	8
Mtb NR	3116	Plasma	3, 8
	M [7]	Peripheral LN (L ing), Thoracic LN [2] (R cran HLN, R carin **gran**), Lung (LUL)	8
Mtb NR	3216	Plasma	3,8
	M [7]	Peripheral LN (R ing), Thoracic LN [2] (R cran HLN,R cran HLN **gran**), Lung (RML)	9
Mtb R	16314	Plasma	3, 8
	M [8]	Peripheral LN (L ing), Thoracic LN [3] (L cran HLN, R cran, HLN, R carin **gran**), Lung [4] (RLL 1 **gran**, RLL 2 **gran**, RLL 3 **gran**, RLL 4 **gran**)	8
Mtb R	20615	Plasma	3, 6
	M [6]	Peripheral LN (R ing), Thoracic LN [2] (**L cran HLN, C carin gran**), Lung [5] (LML, RLL 1 gran, RLL 2 gran, RLL 3 gran, RLL 4 **gran**)	6
Mtb R	31316	Plasma	5, 8
	M [7]	Peripheral LN (R ing), Thoracic LN [2], (L cran HLN, L carin gran), Lung [2] (RUL 1, RUL 2)	8
Mtb R	3516	Plasma	3.8
	M [7]	Peripheral LN (L ing), Thoracic LN [2] (L cran HLN, R carin gran), Lung [4] (RLL 1 **gran**, RLL2 **gran**, RLL3 **gran**, RLL4 **gran**)	9

Table S2. **SIV RNA copy numbers and CD4+ T-cell information for samples used in this study.** Data were previously published in [1]. For plasma RNA, unit is copies/mL, and for tissue RNA, copies/g tissue. 'CD4 count' is the number of CD4+ T cells per g excised tissue or CD4+ T cells per ml of blood for plasma samples,, and 'CD4 percent' is calculated as % of CD3+ T cells. Plasma_nex = plasma taken at necropsy, pLN = peripheral lymph node, tLN = thoracic lymph node.

Cohort	Animal ID	Tissue	Granuloma	RNA	CD4 count	CD4 percent
SIV only	3316	Plasma_nex (Wk 8)	NA	48479	7.9x10^2	35%
SIV only	3316	pLN	N	92100775.2	NA	NA
SIV only	3316	tLN	N	47911290	NA	NA
SIV only	3616	Plasma (Wk 2)	NA	4290572	6.8x10^2	53%
SIV only	3616	Plasma_nex (Wk 8)	NA	1649	1.8x10^2	46%
SIV only	3616	pLN	N	13750750	NA	NA
SIV only	3616	tLN	N	19188847.1	1.3x10^6	42%
SIV only	3816	Plasma_nex (Wk 8)	NA	52156	3.0x10^2	34%
SIV only	3816	pLN	N	25713835.9	1.1x10^6	54%
SIV only	3816	tLN	N	450409973	4.5x10^6	42%
SIV only	3816	Lung	N	817675.367	7.3x10^6	52%
SIV only	4016	Plasma_nex (Wk 8)	NA	20623131	7.5x10^2	46%
SIV only	4016	pLN	N	1357765.83	1.0x10^7	55%
SIV only	4016	tLN	N	284326525	NA	NA
SIV only	4016	Lung	N	25222941.4	8.3x10^5	22%
Mtb NR	30816	Plasma (Wk 5)	NA	539759	1.0x10^3	31%
Mtb NR	30816	Plasma_nex (Wk 8)	NA	2329	2.6x10^3	40%
Mtb NR	30816	pLN	N	28324021.2	NA	NA
Mtb NR	30816	tLN	N	14676594	NA	NA
Mtb NR	30816	tLN	Y	644645652	3.5x10^6	37%
Mtb NR	30816	tLN	Y	13186.5587	1.4x10^5	34%
Mtb NR	3116	Plasma (Wk 3)	NA	2130503	1.2x10^3	35%
Mtb NR	3116	Plasma_nex (Wk 8)	NA	38969	2.3x10^3	44%
Mtb NR	3116	pLN	N	129406528	NA	NA
Mtb NR	3116	tLN	Y	8945666.15	2.5x10^6	50%
Mtb NR	3116	tLN	N	63945105.5	3x10^6	50%
Mtb NR	3116	Lung	N	44935.9131	NA	NA
Mtb NR	3216	Plasma (Wk 3)	NA	22625	2.9x10^2	28%
Mtb NR	3216	Plasma_nex (Wk 8)	NA	9052	3.8x10^2	27%
Mtb NR	3216	pLN	N	12331444.7	NA	NA
Mtb NR	3216	tLN	Y	34823368.8	4.8x10^6	34%
Mtb NR	3216	tLN	N	2088118.24	7.2x10^6	44%
Mtb NR	3216	Lung	N	2140657.97	NA	NA
Mtb R	16314	Plasma (Wk 3)	NA	3487532	7.1.x10^2	12%
Mtb R	16314	Plasma_nex (Wk 8)	NA	52081	8.0x10^1	2%
Mtb R	16314	pLN	N	3763036.06	NA	NA
Mtb R	16314	tLN	N	20723316.4	NA	NA
Mtb R	16314	tLN	Y	17846225.8	NA	NA
Mtb R	16314	tLN	N	1391359.77	1.6x10^6	43%
Mtb R	16314	Lung	Y	300526.001	1.2x10^4	27%
Mtb R	16314	Lung	Y	162548.145	2.2x10^4	21%

Mtb R	16314	Lung	Y	280136.503	3.3x10^6	39%
Mtb R	16314	Lung	Y	280110	9.1x10^4	36%
Mtb R	20615	Plasma (Wk 3)	NA	18965	6.1x10^2	28%
Mtb R	20615	Plasma_nex (Wk 6)	NA	13857658.3	6.1x10^2	23%
Mtb R	20615	pLN	N	33089916.3	NA	NA
Mtb R	20615	tLN	N	3138975.71	NA	NA
Mtb R	20615	tLN	Y	88953205.9	1.9x10^6	44%
Mtb R	20615	Lung	Y	676400.659	2.8x10^3	15%
Mtb R	20615	Lung	N	1085908.58	NA	NA
Mtb R	20615	Lung	Y	16104605	1.4x10^2	13%
Mtb R	20615	Lung	Y	768867.973	1.410^4	47%
Mtb R	20615	Lung	Y	9659220	1.7x10^3	13%
Mtb R	31316	Plasma (Wk 5)	NA	185390	1.5x10^3	24%
Mtb R	31316	Plasma_nex (Wk 8)	NA	209057281	7.5x10^2	43%
Mtb R	31316	pLN	N	51367440.1	NA	NA
Mtb R	31316	tLN	Y	6289421.6	4.9x10^6	51%
Mtb R	31316	tLN	N	552662.451	1.3x10^5	71%
Mtb R	31316	Lung	Y	1576303.7	5.0x10^4	14%
Mtb R	31316	Lung	Y	184405.704	6.6x10^3	10%
Mtb R	3516	Plasma (Wk 3)	NA	141940.09	1.3x10^3	32%
Mtb R	3516	Plasma_nex (Wk 8)	NA	384549.048	1.8x10^3	30%
Mtb R	3516	pLN	N	6349	NA	NA
Mtb R	3516	tLN	N	2353	3.0x10^5	44%
Mtb R	3516	tLN	Y	25655606.3	8.2x10^6	33%
Mtb R	3516	Lung	Y	11017665.6	4.9x10^3	9%
Mtb R	3516	Lung	Y	159054.165	2.5x10^3	7%
Mtb R	3516	Lung	Y	27690.8508	0	0%
Mtb R	3516	Lung	Y	72169.0894	9.5x10^3	10%

Table S3. **Results from the generalized linear mixed model on SIV diversity.** 'Effect' represents the effect size of each factor in relative to SIV only, early plasma samples (i.e. intercept represents SIV only early plasma).

	Estimate	Std. Error	z value	Pr (>|z|)		Effect
(Intercept)	-5.9914763	0.12408955	-48.283489	0	***	0.2%
Week	-0.0511723	0.02908031	-1.7596901	0.07846036	.	-5.0%
Plasma_nex	0.26462795	0.14067385	1.88114526	0.05995216	.	30.3%
pLN	0.22632023	0.14527513	1.55787319	0.11926331		25.4%
tLN	0.32182105	0.14206017	2.26538546	0.02348904	*	38.0%
Lung	0.26150845	0.14367877	1.82009111	0.06874513	.	29.9%
Mtb NR	0.17938493	0.09269056	1.93530964	0.05295231	.	19.6%
Mtb R	-0.0197875	0.0859491	-0.2302239	0.81791783		-2.0%

Table S4. SIV Env substitutions observed in this study that were previously reported [2,3].

AA pos (mac239)	AA sub.	# of sample	Tissue	Cohort	Stock (%)	Ave. freq (%)	(min-max)
120	K120E	4 (5.8%)	Thoracic LN, Lung	Mtb R, Mtb NR	(0.09)	0.61	(0.53 - 0.76)
	K120R	9 (13%)	Plasma, Peripheral LN, Thoracic LN, Lung	Mtb R, Mtb NR	(0.14)	5.54	(0.74 – 19.8)
132	T132S	2 (2.9%)	Plasma, Peripheral LN, Thoracic LN, Lung	SIV only, Mtb R	NA	0.85	(0.72 - 0.98)
135	P135T	1(1.4%)	Plasma	Mtb R	NA	0.52	
136	N136H*	1 (1.4%)	Plasma	Mtb R	NA	0.85	
	N136S*	1 (1.4%)	Peripheral LN	Mtb R	NA	3.03	
	N136K*	1 (1.4%)	Plasma	Mtb R	NA	0.60	
138	T138A*	1 (1.4%)	Thoracic LN	SIV only	NA	8.67	
	T138K*	1 (1.4%)	Lung	Mtb R	NA	0.67	
139	S139P	4 (5.8%)	Plasma, Peripheral LN	SIV only, Mtb R	NA	1.10	(0.68 – 1.72)
	S139A	2 (2.9%)	Plasma, Thoracic LN	SIV only, Mtb R	NA	0.76	(0.66 - 0.86)
	S139L	3 (4.3%)	Plasma, Thoracic LN, Lung	SIV only, Mtb R	NA	1.27	(0.65 - 2.49)
198	N198D	3 (4.3%)	Plasma, Lung	SIV only, Mtb NR, Mtb R	(0.20)	1.00	(0.52 - 1.67)
	N198S	6 (8.7%)	Plasma, Peripheral LN, Thoracic LN, Lung	SIV only, Mtb R	(0.23)	1.55	(0.53 - 3.85)
201	D201N	5 (7.2%)	Plasma, Thoracic LN, Lung	SIV only, Mtb NR, Mtb R	(0.06)	0.95	(0.51 - 1.93)
	D201G	3 (4.3%)	Plasma, Thoracic LN	SIV only, Mtb R	(0.21)	0.82	(0.52 – 1.07)
202	N202D	1 (1.4%)	Plasma	SIV only	(0.07)	1.67	
	N202S	3 (4.3%)	Plasma,Thoracic LN, Lung	SIV only, Mtb NR	(0.09)	2.30	(0.50 - 5.63)
	N202K	3 (4.3%)	Plasma	SIV only	(0.01)	1.22	(0.55 – 1.52)

* indicates a different ancestral amino acid in SIV_{mac251} stock than SIV_{mac239}. The cutoff frequency was 0.5%.

References:

1. Diedrich, C.R.; Rutledge, T.; Maiello, P.; Baranowski, T.M.; White, A.G.; Borish, H.J.; Karell, P.; Hopkins, F.; Brown, J.; Fortune, S.M.; et al. SIV and Mycobacterium Tuberculosis Synergy within the Granuloma Accelerates the Reactivation Pattern of Latent Tuberculosis. *PLoS Pathog* **2020**, *16*, e1008413, doi:10.1371/journal.ppat.1008413.
2. Buckley, K.A.; Li, P.-L.; Khimani, A.H.; Hofmann-Lehmann, R.; Liska, V.; Anderson, D.C.; McClure, H.M.; Ruprecht, R.M. Convergent Evolution of SIV Env after Independent Inoculation of Rhesus Macaques with Infectious Proviral DNA. *Virology* **2003**, *312*, 470–480, doi:10.1016/s0042-6822(03)00262-9.
3. Ita, S.; Hill, A.K.; Lam, E.C.; Dufort, F.J.; Yang, X.; Newman, R.; Leviyang, S.; Fofana, I.B.; Johnson, W.E. High-Resolution Sequencing of Viral Populations during Early Simian Immunodeficiency Virus Infection Reveals Evolutionary Strategies for Rapid Escape from Emerging Env-Specific Antibody Responses. *Journal of Virology* **2018**, *92*, 499–29, doi:10.1128/JVI.01574-17.
